# Nanotechnology-driven strategies in postoperative cancer treatment: innovations in drug delivery systems

**DOI:** 10.3389/fphar.2025.1586948

**Published:** 2025-04-30

**Authors:** Jun-Jie Zhou, Yan-Chuan Feng, Min-Long Zhao, Qi Guo, Xi-Bo Zhao

**Affiliations:** The Stomatological Hospital, Anyang Sixth People’s Hospital, Anyang, China

**Keywords:** local drug delivery system, nanomedicines, postoperative treatment, anti-cancer, combination therapies

## Abstract

Cancer remains a global health challenge, and this challenge comes with a significant burden. Current treatment modalities, such as surgery, chemotherapy, and radiotherapy, have their limitations. The emergence of nanomedicines presents a new frontier in postoperative cancer treatment, offering potential to inhibit tumor recurrence and manage postoperative complications. This review deeply explores the application and potential of nanomedicines in the treatment of cancer after surgery. In particular, it focuses on local drug delivery systems (LDDS), which consist of *in situ* injection, implantation, and spraying. LDDS can provide targeted drug delivery and controlled release, which enhancing therapeutic efficacy. At the same time, it minimizes damage to healthy tissues and reduces systemic side effects. The nanostructures of these systems are unique. They facilitate the sustained release of drugs, prolong the effects of treatment, and decrease the frequency of dosing. This is especially beneficial in the postoperative period. Despite their potential, nanomedicines have limitations. These include high production costs, concerns regarding long-term toxicity, and complex regulatory approval processes. This paper aims to analyze several aspects. These include the advantages of nanomedicines, their drug delivery systems, how they combine with multiple treatment methods, and the associated challenges. Future research should focus on certain issues. These issues are stability, tumor specificity, and clinical translation. By addressing these, the delivery methods can be optimized and their therapeutic efficacy enhanced. With the advancements in materials science and biomedical engineering, the future design of LDDS is set to become more intelligent and personalized. It will cater to the diverse needs of clinical treatment and offer hope for better outcomes in cancer patients after surgery.

## 1 Introduction

Cancer, a disease that has been plaguing humanity, continues to represent a significant challenge to global health. (Cronin et al., 2022; Jassim et al., 2023; Mahalingam and Newsom-Davis, 2023; Bray et al., 2024). The International Agency for Research on Cancer (IARC), through its Global Cancer Observatory, provides a crucial view of the present global cancer situation. (Bray et al., 2024). The latest data in 2022 reveals a reality: t there are almost 20 million new cancer cases emerging, and nearly 10 million lives are unfortunately lost to cancer. (Bray et al., 2024). These figures not only highlight the unyielding rise in the global cancer burden but also underscore the pressing need for comprehensive cancer control strategies (Dhas et al., 2024; Li Q. et al., 2024; Taranto et al., 2024). China, with its extensive population and evolving healthcare dynamics, bears a significant share of this burden. In collaboration with the IARC, the National Cancer Center (NCC) of China has reported that in 2022, the country faced approximately 4.82 million new cancer cases and 2.57 million cancer-related deaths (Bray et al., 2024). These statistics translate to a substantial proportion of the global cancer incidence and mortality rates, with lung, colorectal, thyroid, liver, and stomach cancers being the most frequently diagnosed, and lung, liver, stomach, colorectal, and esophageal cancers leading as the primary causes of cancer mortality (Cronin et al., 2022; Labrie et al., 2022; Bray et al., 2024; Budczies et al., 2024; Lasser et al., 2024).

Currently, the primary treatment modalities for cancer include surgery, chemotherapy, radiotherapy, and immunotherapy (Lim et al., 2018; Cramer et al., 2019; Chow and Longo, 2020). Surgical resection remains the predominant treatment strategy for most solid tumors in clinical practice (Schröder et al., 2021; Mi et al., 2023). Despite the remarkable progress in surgical techniques in recent years, however, minuscule tumor cells may still remain at the surgical margins, significantly increasing the risk of tumor recurrence and metastasis and being closely associated with a diminished overall survival rate (Cannon et al., 2017; Hiller et al., 2017; Tohme et al., 2017; Joshi and Badgwell, 2021).

As for chemotherapy and radiotherapy, which are often employed as adjuvant treatments after surgery, they bring about a host of side effects (Bu et al., 2019; Ji et al., 2020; McLaughlin et al., 2020). Immunosuppression renders patients more susceptible to infections and can delay the recovery process (Ji et al., 2020; McLaughlin et al., 2020).

In this context, the utilization of nanomedicines and controllable drug delivery systems implanted in the surgical region emerges as a highly promising strategy (Wang K-N. et al., 2024). Nanomedicines have the potential to inhibit local tumor recurrence and distant metastasis after surgery. Moreover, they can also deal with postoperative complications. (Shao et al., 2018; Chu et al., 2021; Yang X. et al., 2021; Cheng et al., 2022; Li et al., 2022; Wang et al., 2023; Wang R. et al., 2024; Wei et al., 2024). Nanomedicines provide multiple advantages. These advantages include enhanced targeting capabilities, which allow for a more accurate delivery of drugs to cancer cells and minimize the damage to healthy tissues. (Shi et al., 2016; Zhou et al., 2021; Li ZZ. et al., 2024; Pan et al., 2024). This can be particularly beneficial during the postoperative period. It enables a more sustained treatment approach over time. Moreover, nanomedicines have the potential to target and reduce inflammation at the surgical site. This helps to prevent wound infections and promote the healing process. (Siemer et al., 2019; Jiang et al., 2020; Huang et al., 2024; Zhen et al., 2024).

However, nanomedicines also have some limitations (Shi et al., 2016). The synthesis and formulation of nanomedicines are complex, and this complexity can result in high production costs. (Shi et al., 2016). Besides, there are concerns regarding their long-term toxicity and the potential for accumulation within the body. (Shi et al., 2016). Additionally, the regulatory approval process for nanomedicines tends to be long and filled with challenges.

This paper focuses on exploring the application and potential of nanomedicines in postoperative cancer treatment. It aims to analyze the advantages of nanomedicines, their drug delivery systems, multiple treatment methods, and the associated challenges. The goal is to provide new insights and strategies. These can enhance the effectiveness of cancer treatment, reduce the recurrence risk, minimize postoperative complications, and improve the quality of life of cancer patients.

In the field of cancer treatment, LDDS of nanomedicines have emerged. These methods are a highly promising approach for postoperative therapy. (Zhang et al., 2022; Mimansa et al., 2024). The LDDS has a host of unique advantages. These advantages are crucial for enhancing the effectiveness and safety of cancer treatment after surgery. (Zhang et al., 2022; Mimansa et al., 2024).

First and foremost, when compared with systemic drug delivery, local delivery significantly increases the drug dose in the surgical area (Wang et al., 2023; Lin et al., 2024). This targeted approach ensures a higher concentration of therapeutic agents directly at the site where these agents are most needed. As a result, this maximizes the potential for eliminating residual cancer cells. (Chao et al., 2023; Cao et al., 2024; Wei et al., 2024). For example, in the cases of solid tumors, a concentrated dose of nanomedicines in the surgical bed can be highly effective. This effectiveness is shown in contending with any tumor cells that might have remained after the main tumor is removed. (Erthal et al., 2023; Lin et al., 2024; Wang R. et al., 2024).

Secondly, LDDS can decrease the toxic and side effects of drugs on other organs and tissues. (Li et al., 2021; Liu et al., 2021; Dang et al., 2022). By restricting the drugs to the surgical area, the risk of systemic toxicity is minimized (Zhao et al., 2020). This is especially significant because traditional systemic chemotherapy and radiotherapy frequently lead to a wide variety of adverse effects, including nausea, hair loss, fatigue, and damage to the liver, kidneys, and immune system. (Lu et al., 2021; Guan et al., 2022; Wang et al., 2022). With local nanomedicine delivery, patients can experience fewer side effects and a better quality of life during the recovery period (Xu et al., 2020; Lu Q. et al., 2022).

Moreover, LDDS facilitate the continuous and controllable release of drugs (Wang et al., 2022; Wei et al., 2024). Nanoparticles can be engineered to release drugs at a predetermined rate, ensuring a sustained therapeutic effect over an extended period (Liu et al., 2022a; Wang et al., 2023; Lin et al., 2024). This not only decreases the dosing frequency but also offers a more stable and consistent drug concentration level in the target area. For instance, biodegradable nanoparticles are able to slowly degrade and release drugs over a period of time. This maintains an effective drug level and minimizes the risk of overdosage or underdosage simultaneously (Huang et al., 2021; Yang X. et al., 2021; Liu et al., 2022a; Lu Y. et al., 2022).

Finally, LDDS of nanomedicines can facilitate the growth of normal tissues and the healing of wounds in the surgical area. Some nanomedicines can be designed to release growth factors or other bioactive molecules that are capable of stimulating tissue regeneration and repair. This can not only speed up the healing process but also contribute to the restoration of the normal function of the affected area (Lu Y. et al., 2022; Luo et al., 2022).

**FIGURE 1:**
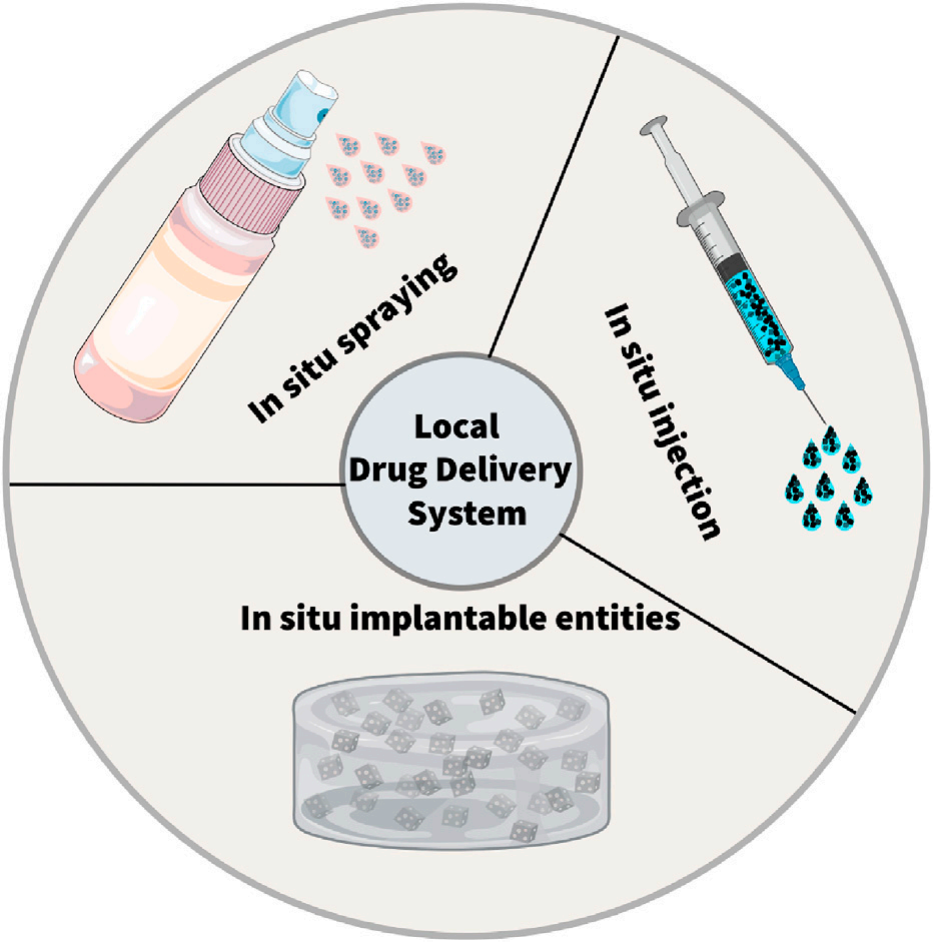
Schematic illustration of the local drug delivery systems (LDDSs). There are three typical strategies within LDDSs, including *in situ* injection, *in situ* implantation, *in situ* spraying.

In this section, we delve deeper into three main LDDS: *in situ* injection, *in situ* implantation, and *in situ* spraying. *In situ* injection involves directly injecting nanomedicines into the surgical site (Zhang H. et al., 2021; Xie et al., 2023; Zhang P. et al., 2024). This method is relatively straightforward. It can be carried out rapidly, enabling the immediate delivery of drugs to the target area. (Figure 1).

**FIGURE 2:**
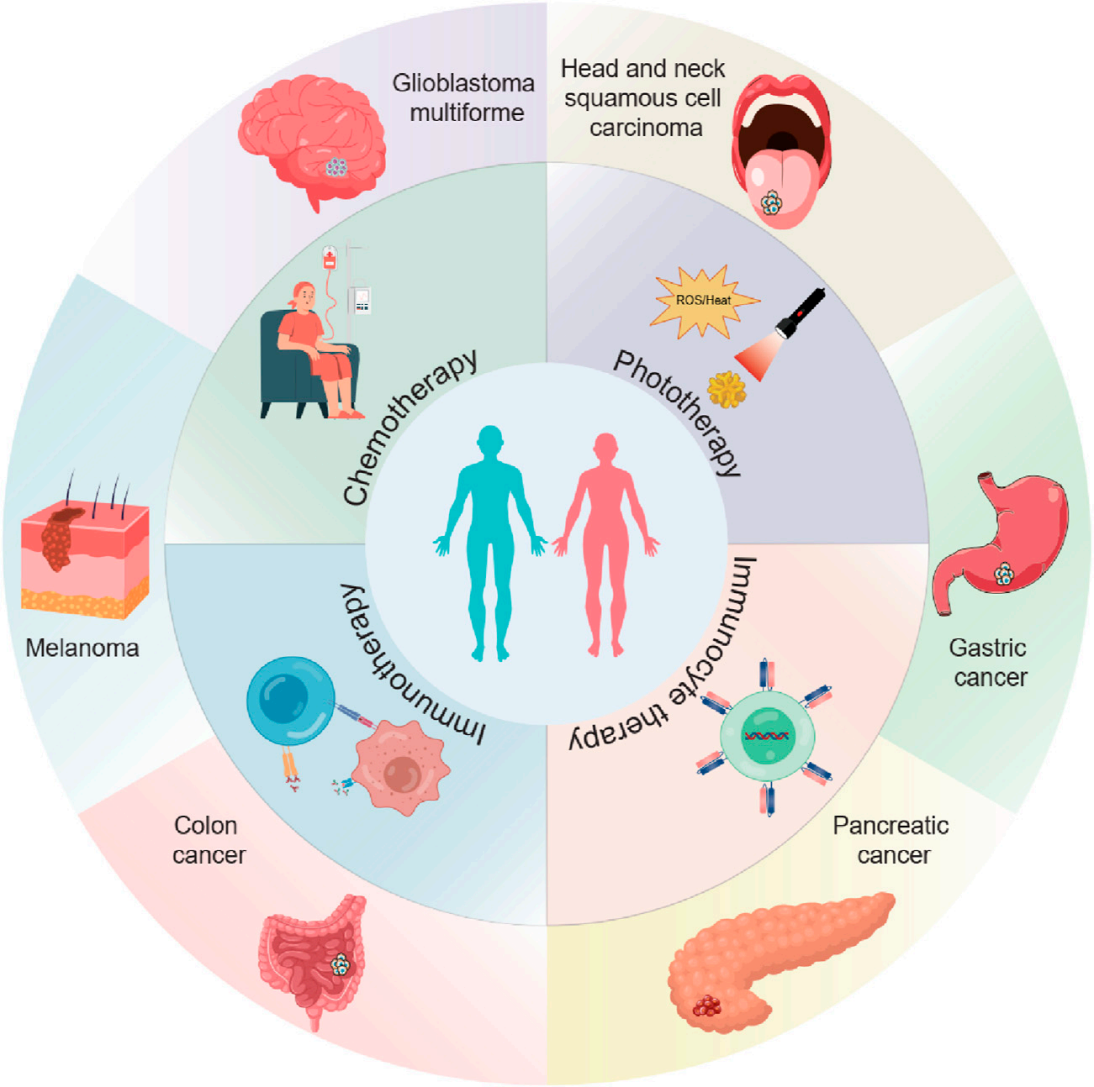
The scheme illustrates the application of local drug delivery systems (LDDSs) in the postoperative treatment of various tumors. The LDDSs are combined with chemotherapy, phototherapy, immunotherapy, and immunocyte therapy.

*In situ* implantation, in contrast, consists of placing an implant loaded with drugs into the surgical area (Hu et al., 2021; Mimansa et al., 2024). This approach can achieve long-term and sustained release of drugs. (Zhou et al., 2021). Biodegradable implants can degrade gradually over time, enabling the controlled release of drugs. *In situ* spraying means spraying a nanomedicine solution onto the surgical wound. (Chen et al., 2018; Shao et al., 2018; Chu et al., 2021). This method can cover the wound surface evenly and ensure a consistent distribution of drugs (Chen et al., 2018; Li et al., 2021). It is especially valuable for large wounds or surgical areas with irregular shapes. Meanwhile, these LDDS offer new hope to patients by cleverly combining multiple advanced treatment methods (Table 1). These methods include chemotherapy, radiotherapy, photothermal and photodynamic therapy, immunotherapy, chimeric antigen receptor T - cell (CAR - T) therapy, and magnetic hyperthermia (Figure 2) (Wu et al., 2018; Zhang et al., 2018; Hu et al., 2021; Li et al., 2021; Wang et al., 2021; Guan et al., 2022; Chao et al., 2023).

**TABLE 1:** Representative nanomaterials summarized in this review for administration strategies of postoperative cancer treatment.

Administration strategies	Materials	In vitro/vivo model	Therapeutics	Reference
*In situ* injection	DOXC_12_-LNC^CL^	GL261 tumor mice model	Chemotherapy	Wang et al. (2023)
	GlioGel	U-87 tumor mice model	Chemotherapy	Erthal et al. (2023)
	DTX-CTs/Gel	B16F10/4T1 tumor mice model	Chemotherapy	Liu et al. (2021)
	GPDF	A375 tumor mice model	Photothermal Therapy	Luo et al. (2022)
	PLCNP	patient-derived GICs tumor mice model	Photodynamic Therapy	Lin et al. (2024)
	Tel@PGE	B16F10/4T1 tumor mice model	Immunotherapy	Liu et al. (2022a)
	THINRTHINR-CXCL10	Luci + GL261^R132H^ tumor mice model	Immunotherapy	Zhang et al. (2021c)
	CAR-T@Met/gel	HGC-27 tumor mice model	Immune cell therapy	Chao et al. (2023)
	GODM-gel	H22 tumor mice model	Immune cell therapy	Cheng et al. (2022)
	GM-CSF	Panc02 tumor mice model	Immune cell therapy	Lu et al. (2022b)
*In situ* implantation	PDA@DH/PLGA	bone tumor mice model	Chemotherapy	Lu et al. (2021)
	TB/αPD-1@AuNCs/OBC	SCC7 tumor mice model	Photothermal Therapy	Zhou et al. (2021)
	Gel-SA-CuO	H22 tumor mice model	Photothermal Therapy	Dang et al. (2022)
	BI(R848 + aOX40)	CT26 tumor mice model	Immunotherapy	Ji et al. (2020)
	3D-ENHANCE-NK cells	MDA-MB-231 tumor mice model	Immune cell therapy	Ahn et al. (2020)
*In situ* spraying	BP@PLEL	HeLa tumor mice model	Photothermal Therapy	Shao et al. (2018)
	GOx@MnCaP@fibrin	IDH1 (R132H) U87 tumor mice model	Starvation/Chemodynamic Therapy	Li et al. (2021)
	aCD47@CaCO3	B16F10 tumor mice model	Immunotherapy	Chen et al. (2018)

In conclusion, LDDS of nanomedicines have significant advantages for postoperative cancer treatment. These systems can provide targeted drug delivery, reduce side effects, facilitate controlled release, and promote tissue healing. Therefore, these methods hold great promise in improving the outcomes of cancer patients after surgery. Furthermore, further research and development in this field are required to perfect these delivery methods and enhance their therapeutic efficacy.

## 2 *In situ* injection

In the field of treatment for tumor recurrence after surgery, the local *in situ* injection drug delivery system offers a brand-new ray of hope to patients deeply afflicted by illness through artfully integrating multiple advanced treatment modalities (Xiong et al., 2021; Zhang J. et al., 2021). Within this diversified treatment landscape, it covers a variety of highly promising means such as chemotherapy, radiotherapy, photothermal and photodynamic therapy, immunotherapy, CAR-T, and magnetic hyperthermia (Yang X. et al., 2021; Luo et al., 2022; Wang et al., 2022; Erthal et al., 2023; Xie et al., 2023; Wei et al., 2024).

### 2.1 Combination of chemotherapy

Local *in situ* injection can accurately deliver chemotherapeutic drugs to the site of tumor recurrence or the surgical area (Erthal et al., 2023; Wang et al., 2023; Wang R. et al., 2024). This targeted approach increases the local drug concentration, enhances the cytotoxic effect of chemotherapy, can more effectively kill residual tumor cells, reduce the risk of tumor recurrence, and simultaneously minimize systemic side effects (Liu et al., 2021; Wang R. et al., 2024).

Glioblastoma multiforme (GBM) is the most common brain tumor and one of the most aggressive cancers in humans (Fiorica et al., 2006; Zhao et al., 2019; Tan et al., 2020; Schaff and Mellinghoff, 2023). Currently, the main clinical treatment for GBM is still surgical resection supplemented by radiotherapy and chemotherapy (Reardon et al., 2006; Roca et al., 2023; Wu and Lim, 2023). However, due to the high permeability of GBM, after the above treatments, tiny tumor cells unavoidably remain in the operative region, ultimately leading to tumor recurrence and metastasis (Zhang W. et al., 2024). As a result, various forms of LDDS *in situ* injection have been used in the treatment of tumor recurrence after surgery.

**FIGURE 3:**
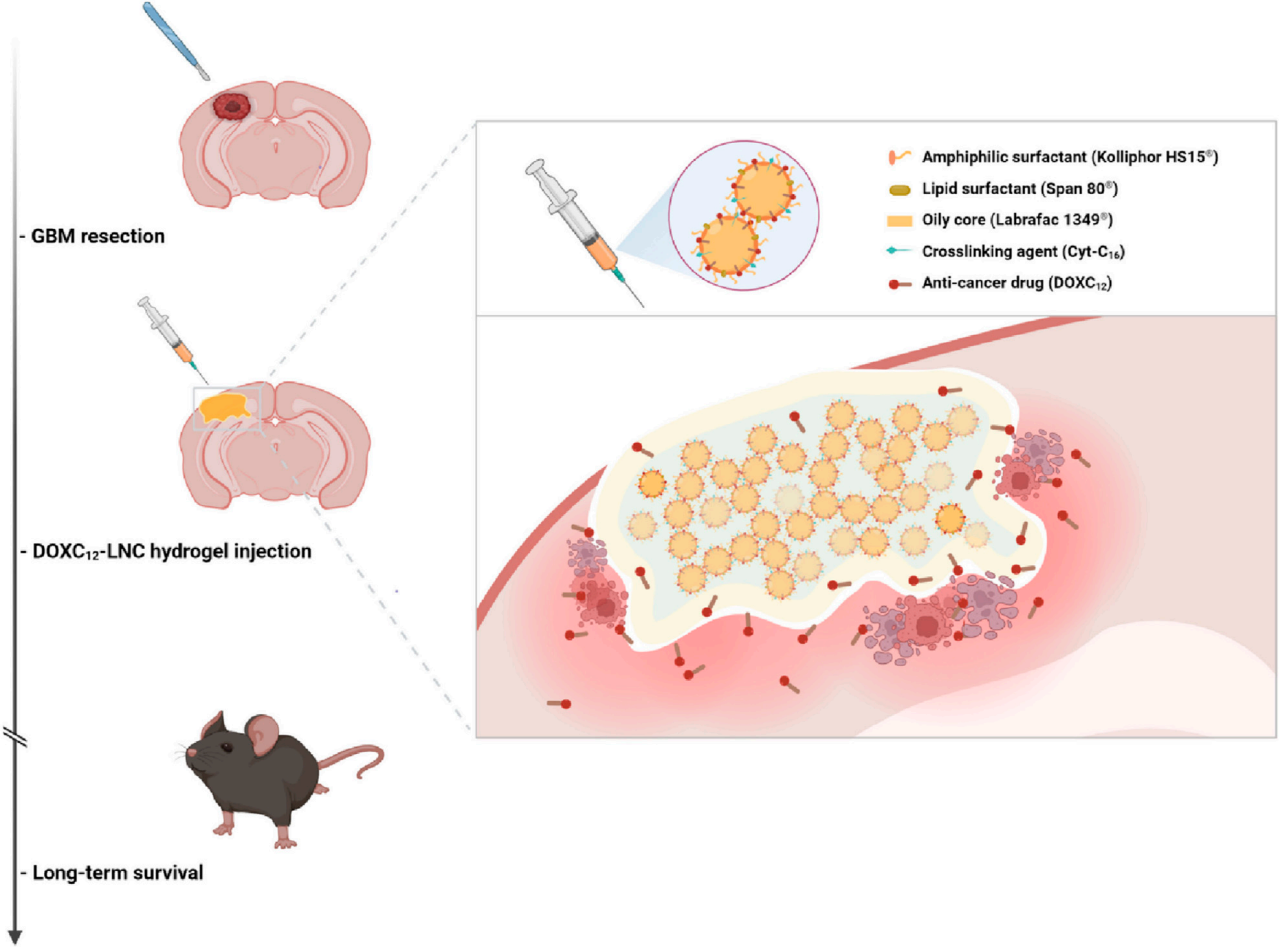
The scheme illustrates the application of DOXC_12_-LNC^CL^ for postoperative local treatment of GBM. Reprinted with permission from Ref (Wang et al., 2023). Copyright 2024 Springer.

Eduardo Ruiz-Hernandez 's research group designed an LDDS (GlioGel), which is composed of an injectable hydrogel carrying free temozolomide and stimulus-responsive paclitaxel-loaded MSN for postoperative local treatment of GBM(Erthal et al., 2023). GlioGel demonstrated a greater ability to penetrate GBM spheroids. The new formulation demonstrated efficacy in decelerating tumor regrowth *in vivo*. It augmented the survival of mice bearing U-87 tumors and enhanced their wellbeing. Simultaneously, Mingchao Wang et al. constructed an injectable lipid nanocapsule (LNC)–based formulation loaded with lauroyl-doxorubicin prodrug (DOXC_12_) (DOXC_12_-LNC^CL^) that integrates the advantages of nanomedicine and local drug delivery to target these infiltrating GBM cells (Figure 3) (Wang et al., 2023). *In vitro* experiments, DOXC_12_-LNC^CL^ showed sustained drug release for more than 1 month, suggesting its potential as a long-term drug delivery system. Moreover, in an orthotopic GL261 GBM preclinical model, the injection of DOXC_12_-LNC^CL^ into the tumor resection cavity resulted significantly inhibiting the recurrence of GBM and prolonging the survival period of mice.

*In situ* injection of LDDS combining chemotherapy are also used in the treatment of melanoma (Liu et al., 2021; Wang R. et al., 2024). Melanoma is a type of skin cancer that develops from the pigment-producing cells called melanocytes (Ajithkumar et al., 2015; Ossio et al., 2017; Centeno et al., 2023). It is often characterized by the uncontrolled growth of abnormal melanocytes, which can form tumors on the skin (Shain and Bastian, 2016; Luke et al., 2017; Carvajal et al., 2023). Melanoma has a risk of tumor recurrence after surgery, especially if it had spread before the operation, and metastasis to other parts of the body may also occur (Lian et al., 2022; Patel et al., 2023). Therefore, surgery plus adjuvant therapy remains the preferred clinical strategy for melanoma (Carvajal et al., 2023; Centeno et al., 2023; Patel et al., 2023). Tianyue Jiang et al. constructed a LDDS (DTX-CTs/Gel) that encapsulates DTX within cell-penetrating peptide-modified transfersomes, followed by embedding them in an oligopeptide hydrogel (Liu et al., 2021). DTX- CTs/Gel can precisely deliver the chemotherapeutic drug DTX to the tumor site, enabling LDDS and increasing the concentration of the drug in the tumor tissue. DTX-CTs has a high skin and tumor penetration efficiency, which can promote the chemotherapeutic drug to cross the skin and penetrate into the tumor tissue, enhancing the therapeutic effect. In mouse melanoma and breast tumor models, DTX-CTs/Gel can effectively slow down tumor recurrence, reduce tumor volume, and improve the therapeutic effect (Liu et al., 2021).

### 2.2 Combination of photothermal and photodynamic therapy

**FIGURE 4:**
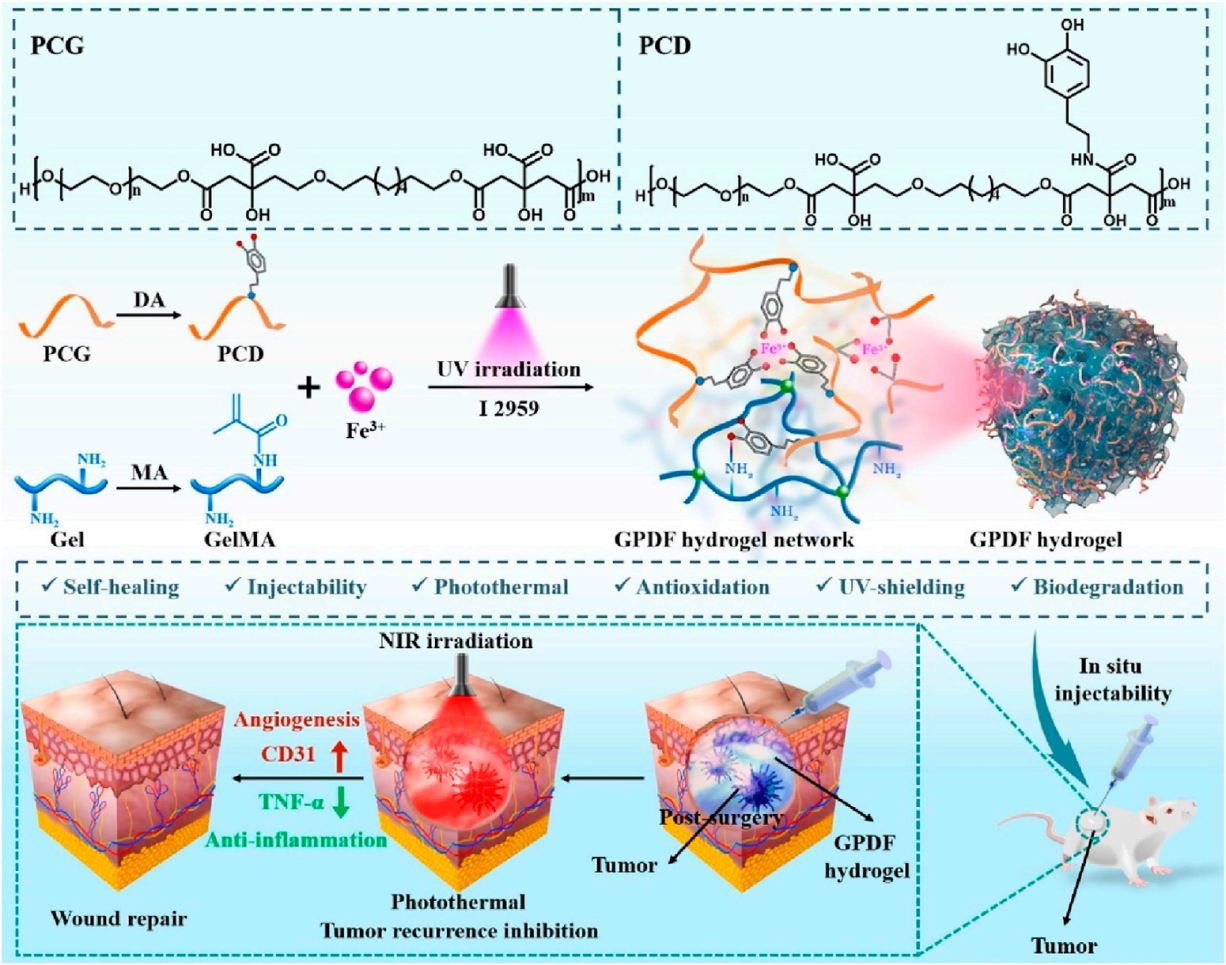
The scheme illustrates a multifunctional photothermal ferric citrate hydrogel scaffold (GPDF) for the treatment of postoperative melanoma. Reprinted with permission from Ref (Luo et al., 2022). Copyright 2022 Elsevier.

Photothermal therapy (PTT), as a minimally invasive treatment method, can convert light energy into heat energy and eliminate tumor tissue through thermal ablation (Li et al., 2020; Xiong et al., 2023; Zhao et al., 2023). Studies have shown that compared with radiotherapy and chemotherapy, PTT is less invasive in the treatment of cancer (Alamdari et al., 2022; Bian et al., 2024). Indocyanine green (ICG) is a near-infrared (NIR) absorbing material widely used in tumor diagnosis and treatment approved by the U.S. Food and Drug Administration (FDA) (Wang et al., 2018; Changalvaie et al., 2019). However, ICG shows limitations such as low light stability, potential toxicity, and poor water stability as a photothermal conversion material (Changalvaie et al., 2019). The new ICG (IR820) has good biocompatibility and stability (Della Pelle et al., 2021; Yang X. et al., 2021; Liu et al., 2024). Therefore, Jinfeng Liao et al. constructed a methylcellulose photothermal hydrogel (IR820/Mgel) for postoperative treatment of breast cancer (Yang X. et al., 2021). The IR820/Mgel hydrogel can quickly heat up under NIR irradiation and can achieve a significant inhibitory effect on tumor recurrence *in vivo* through PTT (Yang X. et al., 2021). At the same time, the IR820/Mgel hydrogel can also achieve breast augmentation by filling the residual cavity after breast surgery and promote breast reconstruction. PTT hydrogels can also be used for postoperative treatment of melanoma. Recently, Lei Bo’s team designed a multifunctional bioactive therapeutic ferric citrate hydrogel scaffold (GPDF) for the treatment of postoperative melanoma (Figure 4) (Luo et al., 2022). The GPDF scaffold has the abilities of injectability, self-healing, antioxidation, enhanced photothermal effect and ultraviolet shielding, and can achieve the purposes of inhibiting tumor recurrence and accelerating wound repair at the same time. The polycitric acid-dopamine (PCD) and Fe^3+^ ions are prepared into a GPDF with a double network through the photo-crosslinking of the gel. Among them, PCD can chelate with Fe^3+^ ions to form dynamic coordination bonds, so that the hydrogel scaffold has injectable and self-healing properties. At the same time, PCD-Fe^3+^ shows excellent photothermal treatment effect and ultra-high efficiency (100%). In conclusion, the GPDF scaffold not only significantly inhibits tumor recurrence but also achieves effective wound repair treatment (Luo et al., 2022). Photodynamic therapy (PDT) indeed shows great promise as a non-invasive cancer treatment approach (Abbas et al., 2017; Yang et al., 2023; Zou et al., 2023). The role of the photosensitizer in PDT is crucial as it facilitates the production of singlet oxygen (Wan et al., 2021; Liu et al., 2022b; Zou et al., 2023). Through the energy transfer between its excited triplet state and the ground triplet state of molecular oxygen, reactive oxygen species (ROS) are generated. These ROS have the ability to selectively target and destroy cancer cells (Wan et al., 2021; Liu et al., 2022b; Zou et al., 2023). Gan Jiang’s research group developed a self - disassembling and oxygen - generating porphyrin - lipoprotein nanoparticle (PLCNP) that can be used for fluorescence - guided surgery and enhanced postoperative PDT in GBM(Lin et al., 2024). The porphyrin - lipoprotein shell enables targeted accumulation of PLCNP in GBM tissue, and the CaO2 core enhances the fluorescence intensity of the porphyrin photosensitizer, improves the imaging effect, and increases the oxygen level and PDT efficiency in GICs(Lin et al., 2024). This nanoplatform prolongs the survival of GICs - bearing mice and can be combined with clinical surgical practices, providing a new strategy for the precise elimination of GBM and future research (Lin et al., 2024).

### 2.3 Combination of immunotherapy

**FIGURE 5:**
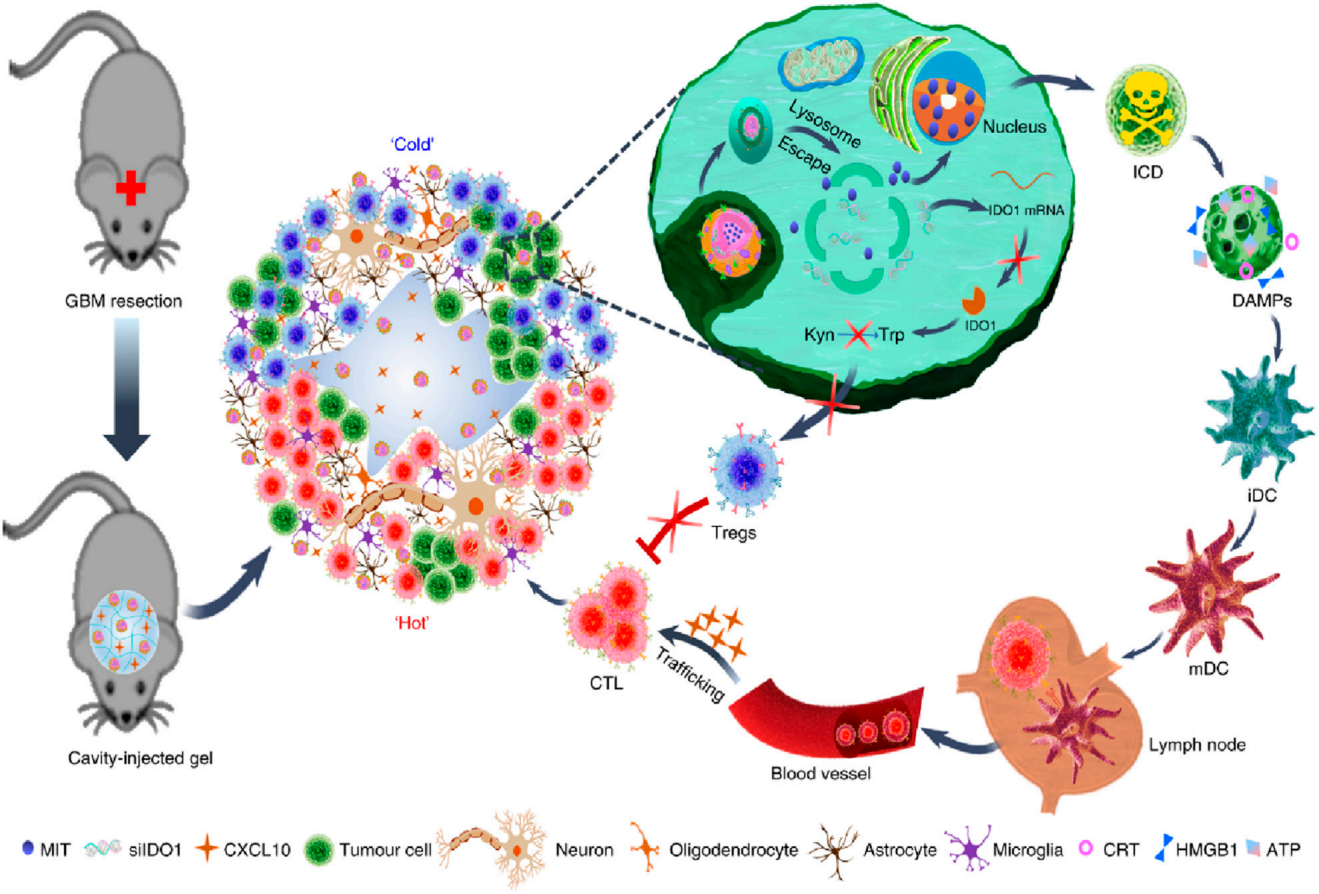
The scheme illustrates an *in situ* self-assembling hydrogel LDDS (THINRTHINR-CXCL10) based on oligopeptides for the treatment of a postoperative mouse model of GBM. Reprinted with permission from Ref (Zhang J. et al., 2021). Copyright 2021 Springer Nature.

Immunotherapeutic agents can also be easily loaded into hydrogels (Yang A. et al., 2021). In recent years, immunotherapy has prevented tumor recurrence after surgery by activating T cells at the tumor site through immune checkpoint blockades (ICB) therapy (Hargadon et al., 2018). However, less than 20% of patients have a sustained clinical response to ICB treatment (Pérez-Ruiz et al., 2020; Schoenfeld and Hellmann, 2020). In addition, ICB is ineffective in tumors characterized by new antigens and somatic mutations. Therefore, it is extremely urgent to relieve the immunosuppressive environment in the tumor microenvironment (TME) (Byun et al., 2017; He and Xu, 2020). Recently, Jiang Xinyi’s research group designed an *in situ* self-assembling hydrogel LDDS (THINRTHINR-CXCL10) based on oligopeptides for the treatment of a postoperative mouse model of GBM (Figure 5) (Zhang H. et al., 2021). The THINRTHINR-CXCL10 hydrogel has the characteristics of high biocompatibility and low viscosity. It is easy to flow during administration and can quickly form a gel network in the cavity of the surgical area. Subsequently, the THINRTHINR-CXCL10 hydrogel acts as a drug reservoir and synergistically releases CXCL10 and THINR in a sustained manner in the surgical area. THINR specifically targets residual tumor cells and is stimulated to decompose under the acidic environment of the TME. The released siIDO1 can relieve Treg-related immunosuppression and activate T cells. At the same time, the CXCL10 chemokine can activate the body’s systemic immunity and enrich T cells to kill residual tumor cells, ultimately significantly inhibiting the recurrence of GBM and prolonging the survival period of mice (Zhang J. et al., 2021). Postoperative recurrence and metastasis of cancer are the main reasons for the high mortality rate. Some immunostimulants can reduce local recurrence and distant metastasis and increase the survival rate during the perioperative period, but excessive immune response is a key therapeutic challenge. Telratolimod (Tel), a TLR 7/8 agonist, has a lipid tail structure to improve hydrophobicity and lymphatic targeting ability, but an effective delivery system is still needed to reduce systemic exposure and inflammatory reactions (Liu et al., 2022a). Liu Hongzhuo developed an injectable delivery platform (Tel@PGE) to deliver Tel to the tumor resection site, which can lead to more infiltration of CD8+T cells and DCs in the tumor tissue and draining lymph nodes, convert M2 macrophages to M1 macrophages, and make the tumor change from “cold” to “hot” and trigger a strong local and systemic immune response at the resection site, inhibit postoperative tumor recurrence and metastasis, improve the survival rate of B16F10 and 4T1 tumor models (Liu et al., 2022a).

### 2.4 Combination of immune cell therapy

Immunocyte therapy is an emerging approach for cancer treatment that utilizes the body’s own immune system to combat tumors (Maxwell et al., 2024; Zeng et al., 2024). This therapy mainly includes cytotoxic T lymphocyte (CTL) therapy, CAR-T therapy, natural killer (NK) cell therapy, tumor-infiltrating lymphocyte (TIL) therapy, and dendritic cell (DC) vaccine therapy, etc., (Maxwell et al., 2024; Zeng et al., 2024). These therapies enhance the recognition and killing ability of immune cells against tumor cells through different means, such as *in vitro* expansion and activation of immune cells, genetic modification of immune cells, and loading of tumor antigens, thereby achieving the goal of treating cancer (Chan et al., 2021; López-Cantillo et al., 2022). Immunocyte therapy has achieved remarkable efficacy in the treatment of some tumors, but further research and development are still needed to improve its therapeutic effect and safety.

**FIGURE 6:**
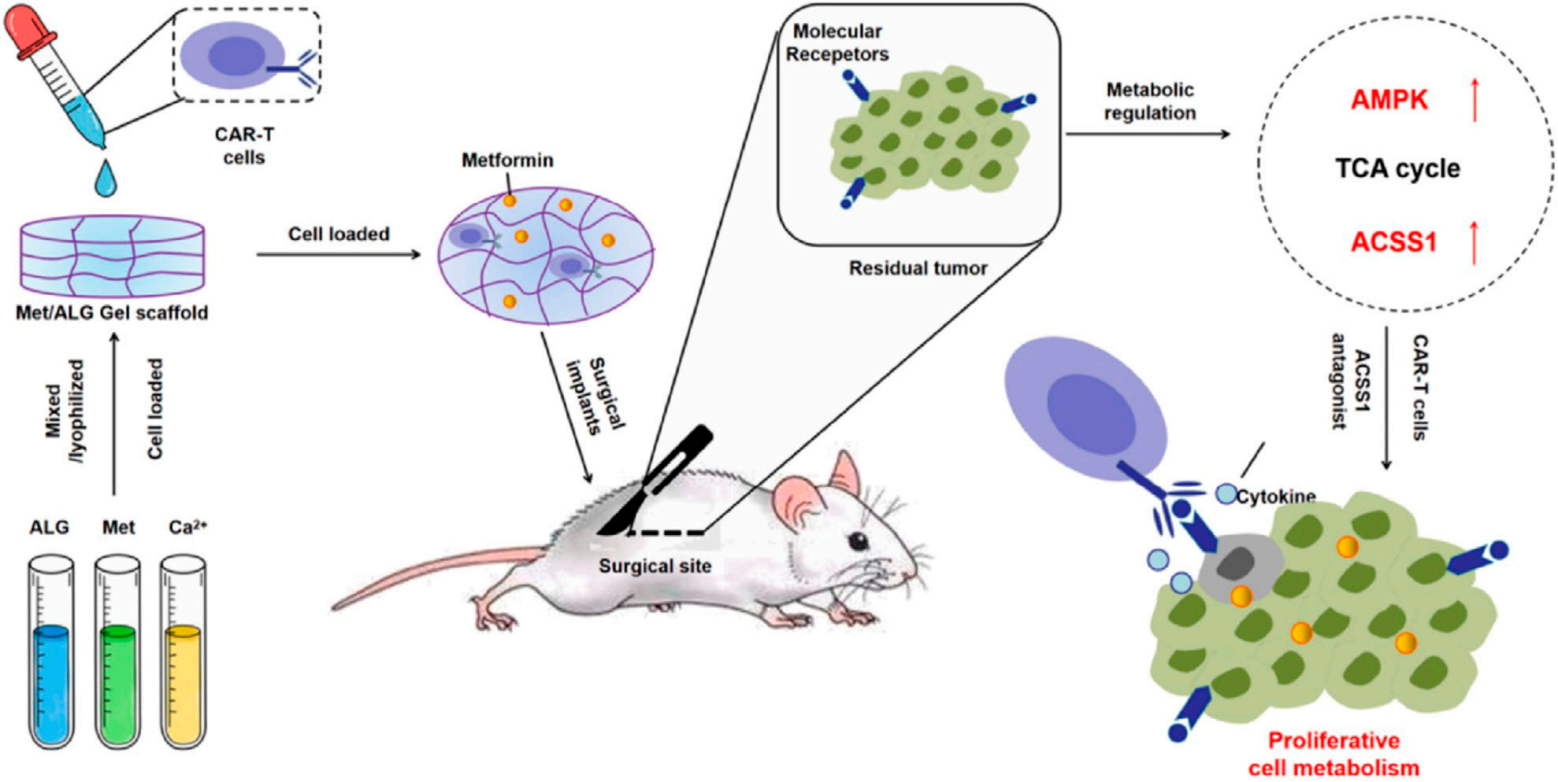
The scheme illustrates a hydrogel scaffold based on sodium alginate to load metformin and CAR-T cells (CAR-T@Met/gel) for the post-surgical tumor models of gastric and pancreatic cancers. Reprinted with permission from Ref (Chao et al., 2023). Copyright 2023 Elsevier.

Studies have shown that *ex vivo* edited T cells are a source of tumor-specific T cells (López-Cantillo et al., 2022). It has been proven that T cells expressing chimeric antigen receptor are particularly effective in the treatment of some patients with malignant hematological tumors (Sterner and Sterner, 2021; López-Cantillo et al., 2022). In contrast, the application of CAR-T cell therapy in solid tumors is still challenging. This may be because the TME in solid tumors has a highly immunosuppressive effect and can induce CAR-T cell exhaustion (Blass and Ott, 2021). Then, combining CAR-T cells and ICB may be one of the ways to improve the role of CAR-T cells in solid tumors (Blass and Ott, 2021). It was found that metformin could upregulate the oxidative phosphorylation and energy metabolism of CAR-T cells, promote their proliferation, and simultaneously inhibit the oxidative and glycolytic metabolism of cancer cells, reducing tumor hypoxia. Thus, Liu Zhuang’s group designed a hydrogel scaffold based on sodium alginate to load metformin and CAR-T cells (CAR-T@Met/gel) (Figure 6) (Chao et al., 2023). CAR-T@Met/gel showed the strongest tumor recurrence prevention effect in the post-surgical tumor models of gastric and pancreatic cancers, and could significantly reduce the tumor volume. Meanwhile, CAR-T@Met/gel has an excellent antitumor response against post-surgical solid tumors with high safety. This strategy provides a modular platform technology for addressing the critical challenge of the ineffectiveness of CAR-T cell therapy against solid tumors and has the potential for clinical translation.

**FIGURE 7:**
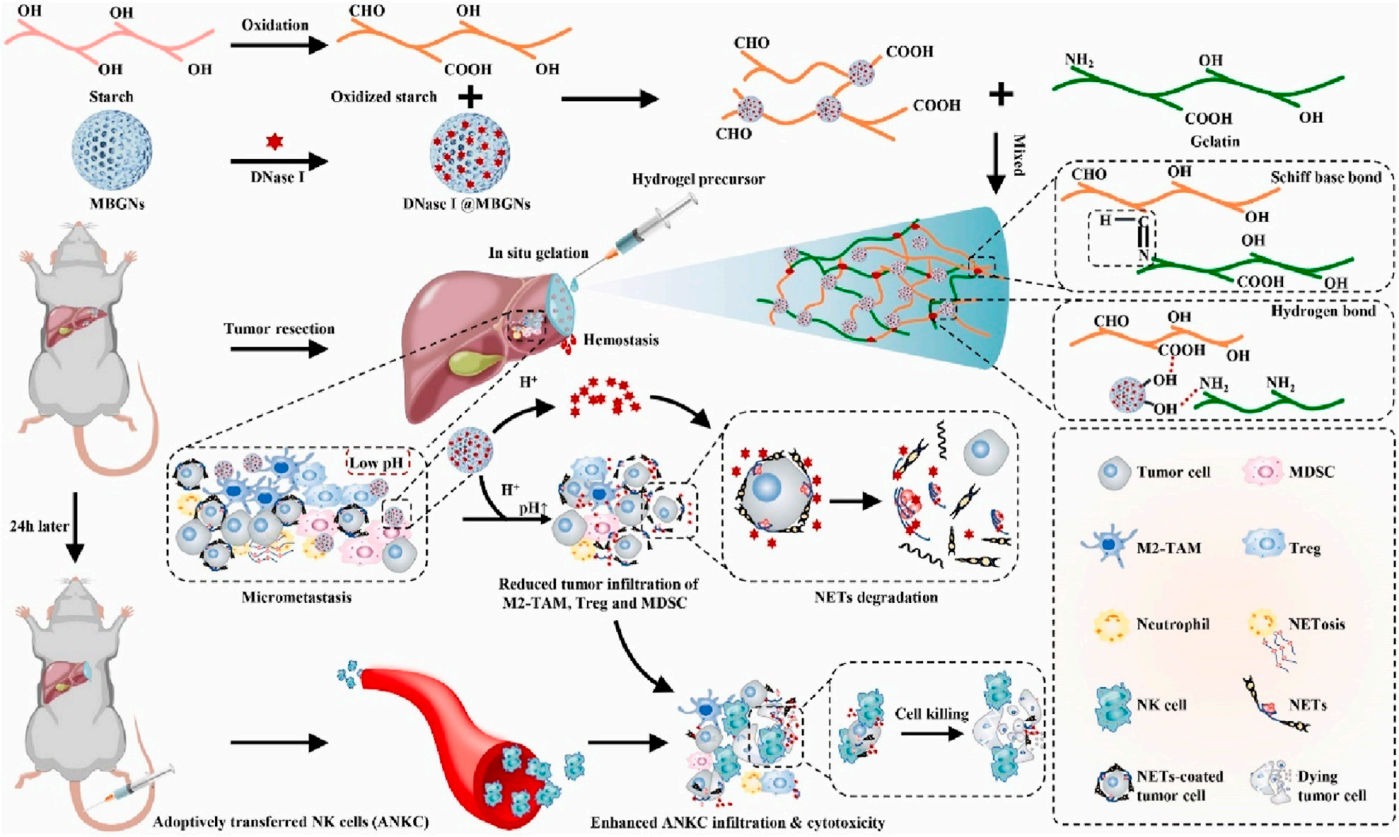
The scheme illustrates an *in situ* injectable dual pH-responsive hydrogel for enhancing adoptive NK cell therapy to prevent post-resection HCC recurrence. Reprinted with permission from Ref (Cheng et al., 2022). Copyright 2022 Elsevier.

NK infusion is considered a promising cancer therapy, but the acidic TME and neutrophil extracellular traps (NETs) greatly weaken its therapeutic effect (Vivier et al., 2008; Dagher and Posey, 2023; Vivier et al., 2024). Wenjie Chen et al. developed a dual pH-responsive hydrogel cross-linked with a tumor acidity neutralizer (mesoporous bioactive glass nanoparticles) and a NET lyase (deoxyribonuclease I, DNase I), and used it in combination with NK infusion to prevent postoperative tumor recurrence (Figure 7) (Cheng et al., 2022). This hydrogel can be injected into the surgical margin and form an adherent gel with a rapid hemostatic effect. At the same time, it neutralizes the acidic environment of TME to reduce tumor infiltration of immunosuppressive cells and releases DNase I under pH response to lyse NET. This combination therapy significantly enhances the therapeutic effect of NK infusion, inhibits postoperative tumor recurrence, and does not produce systemic toxicity.

In addition, more and more evidence show that LDDS can be tailored according to the patient’s condition to promote personalized tumor treatment (Lu Y. et al., 2022). Tumor cells are the main targets of anti-tumor immune responses, but TME will produce a series of immunosuppressive mechanisms during malignant progression to promote tumor immune escape (Saleh and Elkord, 2020). Obtaining relevant biological information through resected surgical specimens can guide the preparation of personalized hydrogel LDDS (Lu Y. et al., 2022). For example, Deng Junjie’s research group developed a hydrogel vaccine system based on granulocyte-macrophage colony-stimulating factor (GM-CSF). The hydrogel is prepared by cross-linking the lysate of surgically resected tumor cells at a low temperature (Lu Y. et al., 2022). The personalized GM-CSF released by the hydrogel vaccine system can recruit DCs, which provides a personalized tumor antigen pool. They combine to promote the activation of the individual immune system. Implanting this personalized hydrogel vaccine system into the surgical area activates a strong anti-tumor immune response in the body and eliminates residual tumor cells after surgery. *In vivo* experiments, this personalized hydrogel vaccine system combined with anti-programmed cell death one ligand 1 (αPD-L1) antibody significantly inhibited tumor recurrence and metastasis in a postoperative tumor model of pancreatic cancer mice.

*In situ* injection has emerged as a promising strategy for postoperative cancer treatment due to its direct delivery of therapeutics to the surgical site. The primary advantages include rapid administration, localized drug accumulation, and minimized systemic toxicity, which collectively enhance therapeutic efficacy while reducing off-target effects (Wang et al., 2023). However, challenges remain. Precise control over drug release kinetics is technically demanding, as uneven distribution or premature degradation may compromise efficacy. Patient-specific factors, including tumor heterogeneity and surgical cavity morphology, may also influence outcomes, highlighting the need for personalized formulations. Addressing these limitations through advanced material engineering and long-term safety studies will be critical to optimizing *in situ* injection for broader clinical adoption.

## 3 *In situ* implantation

*In situ* implantable entities encompass various forms, including implantable hydrogel scaffolds and tissue engineering scaffolds (Sun et al., 2011; Al-Jawadi et al., 2018). These biological entities boast numerous remarkable advantages, regarding biological safety, which lack immunogenicity and adverse reactions towards the human body, and will not trigger human immune rejection reactions, thereby greatly ensuring patient safety (Sun et al., 2011; Al-Jawadi et al., 2018). They exhibit excellent biocompatibility and can coexist harmoniously with human tissues without causing inflammation or other adverse physiological responses (Al-Jawadi et al., 2018). Simultaneously, they also possess excellent biodegradability. After fulfilling their specific therapeutic functions, they can be gradually decomposed and metabolized within the body and will not remain for a long time to cause potential harm to the human body (Al-Jawadi et al., 2018).

Numerous studies have demonstrated that implantable biological entities have been widely utilized in clinical treatments, particularly playing an important role in promoting wound healing and tissue regeneration (Balaure et al., 2019). Wound healing is a complex physiological process. Implantable biological entities can provide a suitable microenvironment for wounds, promote cell proliferation and differentiation, and accelerate the closure and repair of wounds. In terms of tissue regeneration, implantable biological entities can act as scaffolds to guide the growth and development of new tissues and restore the functions of damaged tissues.

### 3.1 Combination of chemotherapy

**FIGURE 8:**
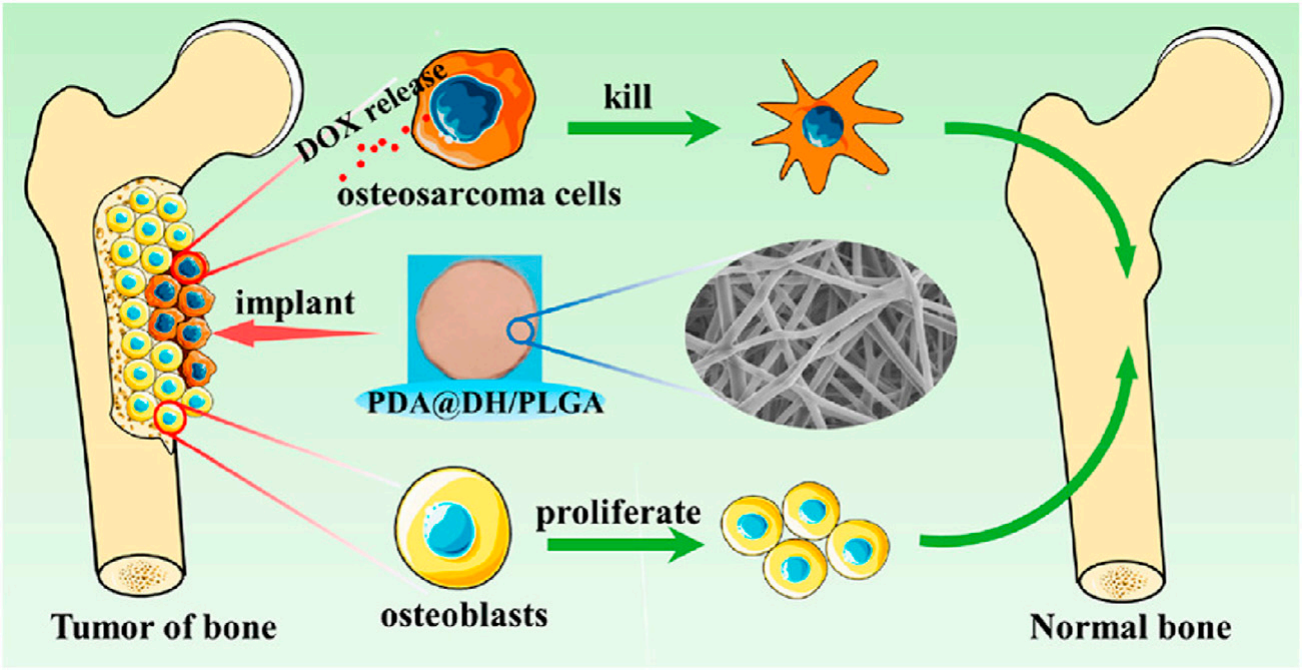
The scheme illustrates a polydopamine (PDA)-coated composite (PDA@DH/PLGA) for simultaneously repairing bone defects and preventing tumor recurrence. Reprinted with permission from Ref (Lu et al., 2021). Copyright 2021 American Chemical Society.

Currently, a variety of implantable biological entities have been applied in post-tumor treatment (Bagó et al., 2016; Zhang H. et al., 2021). For instance, Zhiwei Yang et al. prepared a polydopamine (PDA)-coated composite (PDA@DH/PLGA) (Lu et al., 2021). PDA@DH/PLGA achieved controlled drug release, inhibited the growth of tumor cells in the early stage, promoted the proliferation of normal cells in the later stage, and greatly enhanced the bioactivity. The PDA@DH/PLGA scaffold is expected to be a potential candidate for simultaneously repairing bone defects and preventing tumor recurrence (Figure 8).

### 3.2 Combination of PTT and PDT

**FIGURE 9:**
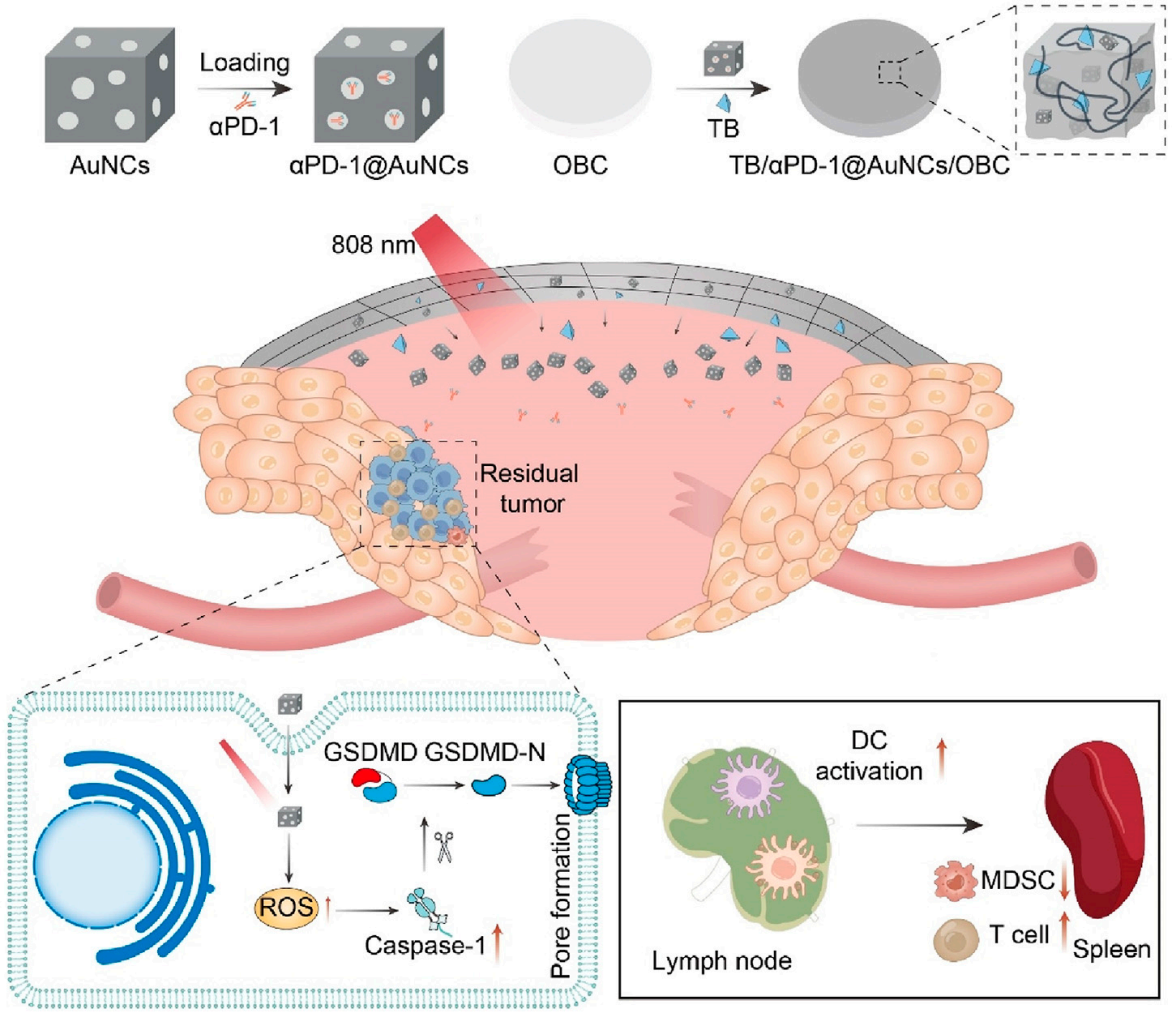
Schematic illustration of the versatile OBC-based membrane for achieving a therapeutic effect in antitumor immunotherapy towards HNSCC postoperative treatment.

The recurrence of head and neck squamous cell carcinoma (HNSCC) following surgical resection remains a significant challenge in cancer treatment (Mahvi et al., 2018; Chow and Longo, 2020). Advanced HNSCC shows a low response rate to ICB, whereas PTT can enhance the infiltration of immune cells, making tumors more receptive to cancer immunotherapy. Our group designed and constructed a novel multifunctional nanocomposite (TB/αPD-1@AuNCs/OBC) consisting of oxidized bacterial cellulose (OBC), thrombin (TB), and gold nanocages (AuNCs) containing anti-programmed death receptor 1 (αPD-1) antibody (αPD-1@AuNCs), enabling the combination of therapies to achieve remarkable postoperative antitumor immunity and control local tumor recurrence (Figure 9) (Zhou et al., 2021). The TB/αPD-1@AuNCs/OBC + L could generate ROS to induce pyroptosis and release intracellular contents, which triggered T-cell-mediated robust tumor eradication due to the enhanced DC process and presentation of tumor-specific antigens.

The TB/αPD-1@AuNCs + L displayed potent photothermal therapeutic effect, which activated potent antitumor immunity combined ICB therapy to prevent tumor recurrence. Meanwhile, the TB/αPD-1@AuNCs/OBC could be adapted in ‘real-world practice’ and might a promising candidate for HNSCC treatment. Furthermore, Mingqiang Li’s group developed an implantable 3D printed hydrogel scaffold (Gel-SA-CuO) that inhibits postoperative tumor recurrence by combining glutathione (GSH) depletion-induced ferroptosis and photothermal-augmented chemodynamic therapy (Dang et al., 2022). They used 3D printing technology to prepare a hydrogel scaffold containing CuO nanoparticles, which allows for controlled and sustained release of CuO during the biodegradation process. CuO nanoparticles function as a reservoir for releasing Cu^2+^ to generate intracellular ROS and also serve as a photothermal agent to generate heat. The heat generated by photothermal conversion further enhances the efficiency of the Fenton-like reaction. Moreover, the scaffolds induce ferroptosis through GSH depletion and inactivation of GPX4. The results indicated that Gel-SA-CuO offered a novel treatment strategy for the inhibition of postoperative tumor recurrence.

### 3.3 Combination of immunotherapy

**FIGURE 10:**
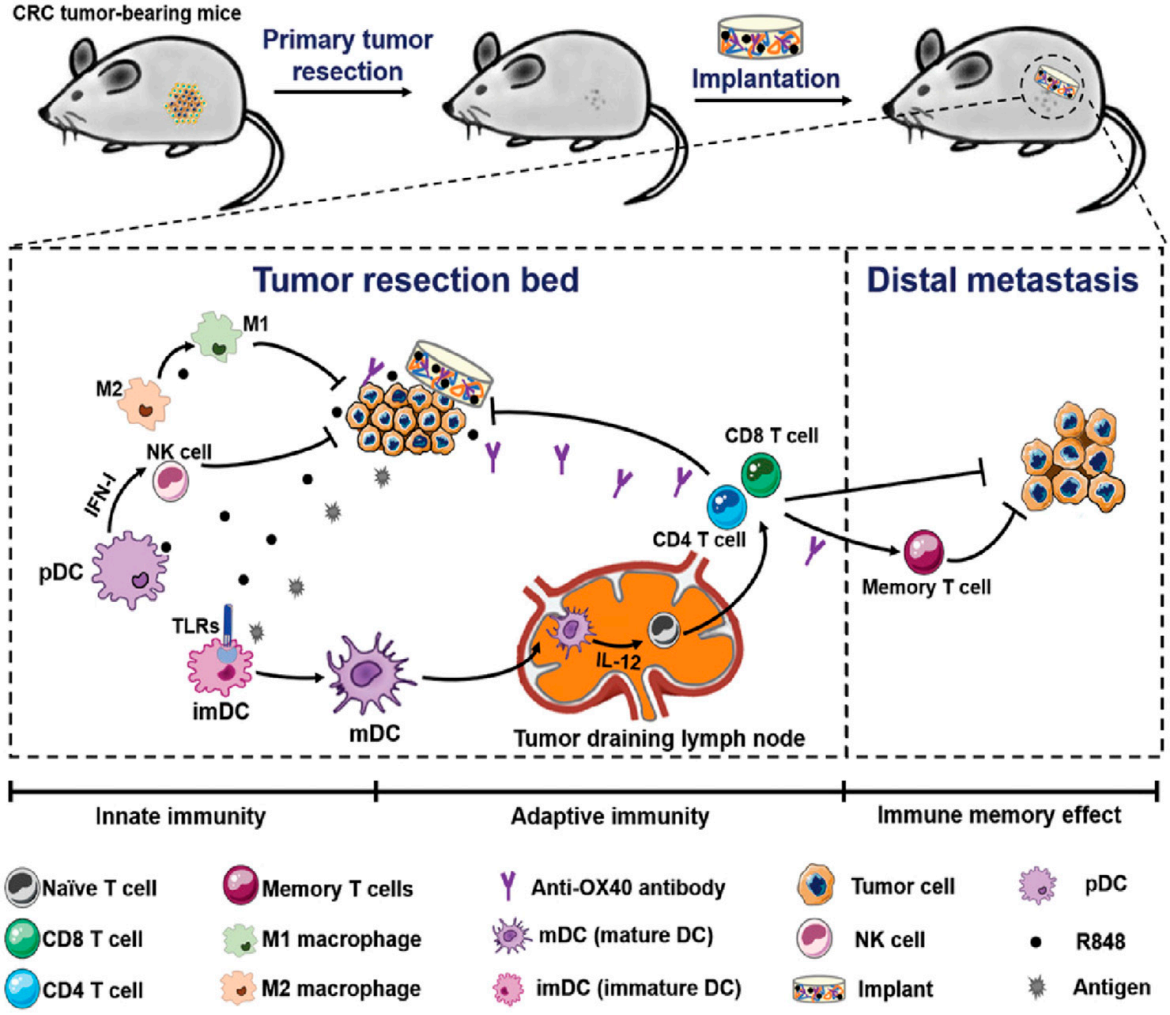
Schematic illustration of the biopolymer immune implant BI(R848 + aOX40) for preventing CRC postoperative tumor relapse and metastasis. Reprinted with permission from Ref (Ji et al., 2020). Copyright 2020 WILEY.

Colorectal cancer (CRC) is a commonly occurring malignant tumor in the digestive tract, and surgery is the first-line treatment for it (Fakih, 2015; Biller and Schrag, 2021; Moorman et al., 2024). However, for advanced CRC, the efficacy of surgical resection is limited, and the recurrence and metastasis of tumors after surgery lead to high morbidity and mortality (Fakih, 2015). Postoperative *in situ* immunotherapy presents a promising option for preventing tumor recurrence and metastasis, and material-based local immunotherapy has potential in cancer treatment. Xuesi Chen et al. proposed a biopolymer implant fabricated with 4-arm poly (ethylene glycol) amine (4-arm PEG-NH2) and oxidized dextran (ODEX), and co-loaded with resiquimod (R848) and anti-OX40 antibody (aOX40) for CRC post-surgical treatment (Figure 10) (Ji et al., 2020). The research team demonstrated a straightforward post-surgical CRC immunotherapy strategy by placing pre-formed therapeutic biopolymer immune implants in the tumor resection cavity. The sequential activation of innate and adaptive immunity, along with immune memory effects from the gradual release of R848 and aOX40, not only resulted in complete clearance of residual tumors but also inhibited the growth of distant tumors and provided resistance to tumor re-challenge.

### 3.4 Combination of immune cell therapy

*In situ* implantable biological entities do indeed include various forms, such as implantable hydrogel scaffolds and tissue engineering scaffolds (Zhang et al., 2023). These entities can provide scaffolding functions for immune cells (Singh and Peppas, 2014). Specifically: Implantable hydrogel scaffolds have good biocompatibility and adjustable physicochemical properties, which can provide a suitable living environment and attachment sites for immune cells (Singh and Peppas, 2014). Tissue engineering scaffolds can simulate the structure and function of the extracellular matrix, guide the migration, proliferation, and differentiation of immune cells, and promote the effective recruitment and activation of immune cells (Singh and Peppas, 2014).

**FIGURE 11:**
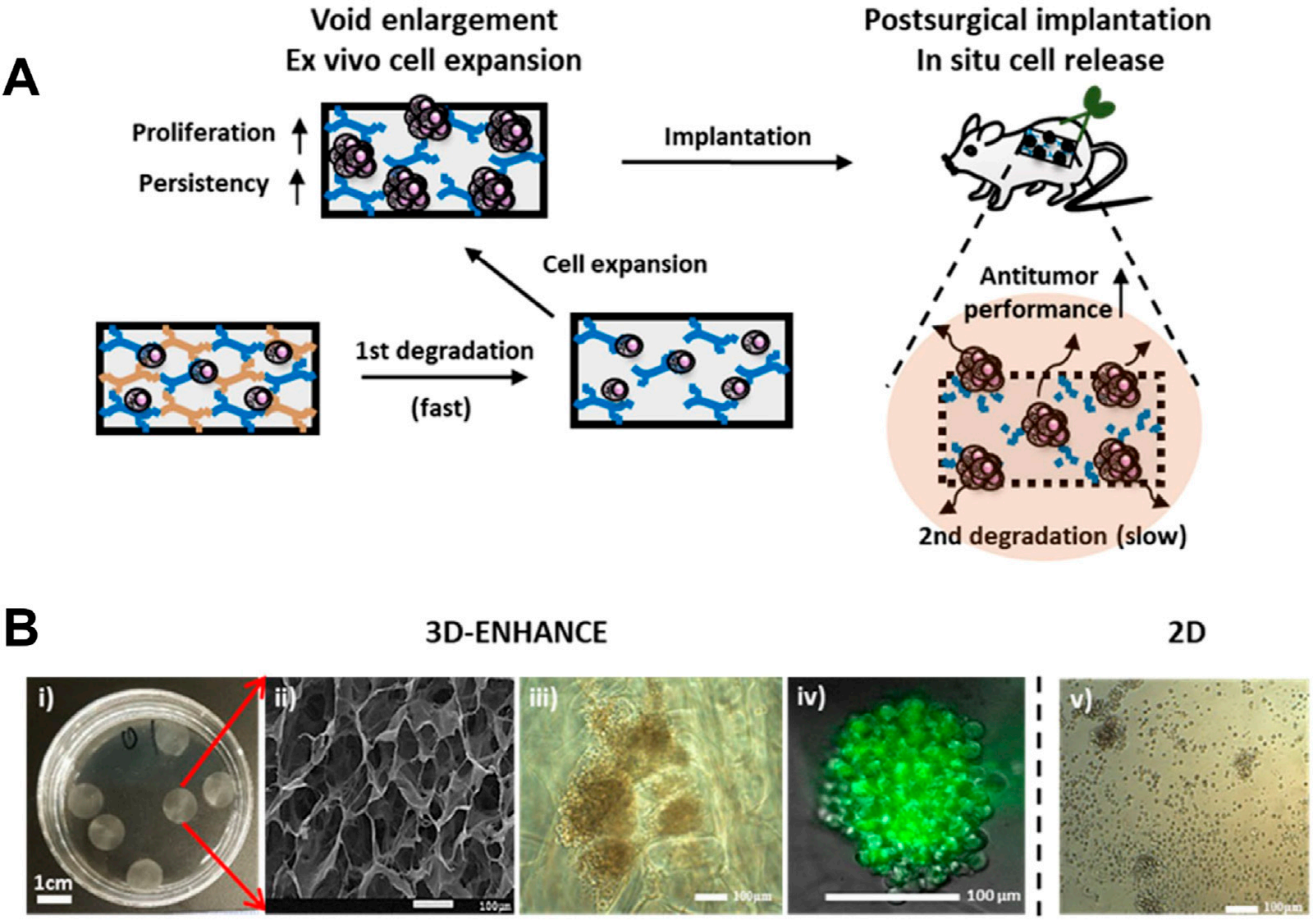
**(A)** Schematic illustration of the 3D-ENHANCE loaded with NK cells for preventing postoperative tumor relapse. **(B)** Macroporous architecture of 3D-ENHANCE. i) Photograph of 3D-ENHANCE. ii) The scanning electronic microscopic image for 3D – ENHANCE. iii) Bright field image of NK cell clusters formed in 3D-ENHANCE. iv) Live cell image of NK cell cluster formed in 3D-ENHANCE. v) Bright field image of NK cell cultured in the 2D manner. Reprinted with permission from Ref (Ahn et al., 2020). Copyright 2020 Elsvier.

For instance, the team led by Tae-Don Kim proposed a 3D multi-polymer scaffold (3D-ENHANCE) constructed based on HA (Figure 11) (Ahn et al., 2020). This scaffold features a unique porous niche-like structure that is highly conducive to cell amplification. It provides a cytokine-free microenvironment for the expansion of NK cells *in vitro*, significantly enhancing the tumor immunotherapeutic efficacy of NK cells. After in-depth research, it was found that 3D-ENHANCE can be rapidly degraded within 18 days. This characteristic avoids the potential risks of long-term residual in the body. At the same time, it also significantly inhibits the recurrence and metastasis of tumors after breast cancer surgery. In the treatment of breast cancer, after surgical resection of the tumor, there is often a risk of residual tumor cells. The application of the 3D-ENHANCE scaffold provides a new solution to reduce this risk.

## 4 *In situ* spraying

The *in situ* spraying drug delivery system presents several remarkable advantages (Chen et al., 2018; Shao et al., 2018; Chu et al., 2021; Li et al., 2021). It often employs a multi-in-one spray bottle to blend liquid gels with multiple functional components and then sprays them onto the wound surface (Chen et al., 2018; Shao et al., 2018; Chu et al., 2021; Li et al., 2021). Taking advantage of the unique physiological environment of the wound, it rapidly organizes and forms a film covering the wound, which is highly suitable for relatively large wound surfaces after tumor resection. This system can extensively cover the entire wound area, playing roles in hemostasis, antibacterial activities, promoting wound healing, and slowly releasing drugs.

In previous studies, Yu Xuefeng’s research group designed a degradable black phosphorus nano-spray photothermal hydrogel (Shao et al., 2018). After being sprayed on the tumor surgical area, this spray hydrogel can quickly form a film. While eliminating residual tumor cells in the surgical area through PTT, it also takes into account the bactericidal effect of PTT, reducing the occurrence of common clinical complications such as wound infection. The biocompatibility and degradability of this photothermal spray hydrogel are excellent, and it can be gradually degraded in the body, facilitating its further transformation towards clinical applications.

**FIGURE 12:**
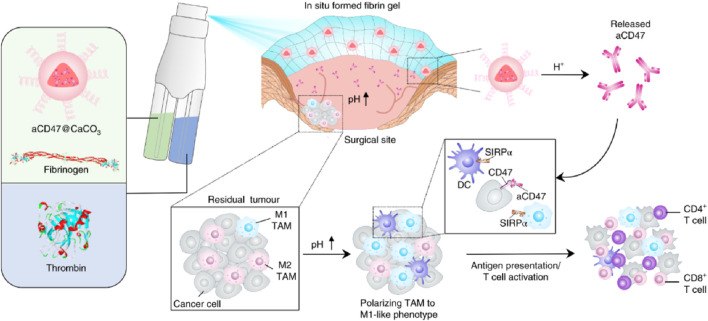
Schematic illustration of the *in situ* sprayed bioresponsive fibrin gel containing aCD47@CaCO_3_ nanoparticles for preventing postoperative tumor local recurrence. Reprinted with permission from Ref (Chen et al., 2018). Copyright 2018 Springer Nature.

In addition, Gu Zhen’s team developed an immunotherapy spray (Figure 12) (Chen et al., 2018). After spraying on the surgical site, it can form a bioactive gel. The immunotherapy antibody embedded in it can be slowly released to awaken the body’s immune system and control postoperative local tumor recurrence and the occurrence of potential distant tumor metastasis. This immunotherapy spray is a two-in-one storage spray device. One storage bottle contains fibrinogen and calcium carbonate nanoparticles loaded with αCD47 antibody, and the other storage bottle contains TB solution. After spraying on the surgical site, TB in the solution quickly acts in the environment of micro-bleeding in the surgical area, making the entire gel adhere to the surface of the surgical area. At the same time, the low pH value in the TME will gradually interact with calcium carbonate nanoparticles and release αCD47 antibody, thereby activating the immune system and clearing residual tumor cells in the surgical area. Later, this research group further tested the *in vivo* effect of the spray gel through a postoperative model of melanoma mice. According to the *in vivo* imaging results, it can be seen that after receiving spray treatment, tumor cells in more than 50% of mice are completely inhibited, significantly improving the survival period of mice.

Approximately 12% of malignant gliomas express the IDH1 gene, and the R132H mutation accounts for 92.7% of IDH1 gene mutations (Choi et al., 2018). IDH1 is a very important enzyme in the tricarboxylic acid cycle. Isocitrate is converted into α-ketoglutaric acid under the catalysis of IDH1, thereby forming nicotinamide adenine dinucleotide phosphate (NADPH). NADPH is a sacrificial agent for intracellular ROS (McBrayer et al., 2018). Therefore, when the IDH1 gene has an R132H mutation, the expression level of NAPDH will be significantly reduced, thereby increasing the sensitivity of IDH1 cells to oxidation. In addition, mutated IDH1 mainly obtains energy through glycolysis (Fu et al., 2015). Therefore, by reducing the glucose level in the postoperative environment and increasing the generation of ROS, it is expected to inhibit the recurrence of IDH1 (R132H) glioma. Based on this, Huang Peng’s team developed a spray gel combining starvation therapy/chemodynamic therapy to inhibit residual IDH1 (R132H) glioma cells after surgery (Li et al., 2021). This team first mineralized glucose oxidase and further loaded it into fibrin gel. When this gel material is sprayed on the surgical area, glucose oxidase can catalyze the oxidation reaction of glucose to produce hydrogen peroxide, which increases the level of ROS while reducing the glucose level in the surgical area environment. The increased ROS further kills the residual IDH1 (R132H) glioma cells, and finally significantly inhibits the recurrence of IDH1 (R132H) glioma. Yao He et al. developed a multifunctional flavonoid-silica nanocomposite (FSiNCs) with good aqueous solubility and fluorescent properties using a facile microwave-assisted synthetic method (Chu et al., 2021). *In vitro* experiments showed that FSiNCs have concentration-dependent cytotoxicity towards cultured cancer cells and normal cells, with higher toxicity towards cancer cells. The developed FSiNCs@Fibrin gel shows potential in preventing post-surgical tumor recurrence and bacterial infections, suggesting its potential for clinical translation in the treatment of post-surgical cancer recurrence and infections.

## 5 Avoid complications after cancer surgery

Postoperative complications of tumors are significant factors that affect the recovery and quality of life of patients (Nathan et al., 2016). Common complications include bleeding, infection, poor wound healing, lymphedema, organ dysfunction, deep vein thrombosis, intestinal obstruction, nerve injury, pain, and tumor recurrence or metastasis (Savioli et al., 2020). These complications are related to various factors such as surgical trauma, decreased immune function, and residual tumor cells (Eto et al., 2017). The LDDS emerges as a novel therapeutic strategy, providing new ideas for resolving postoperative complications.

**FIGURE 13:**
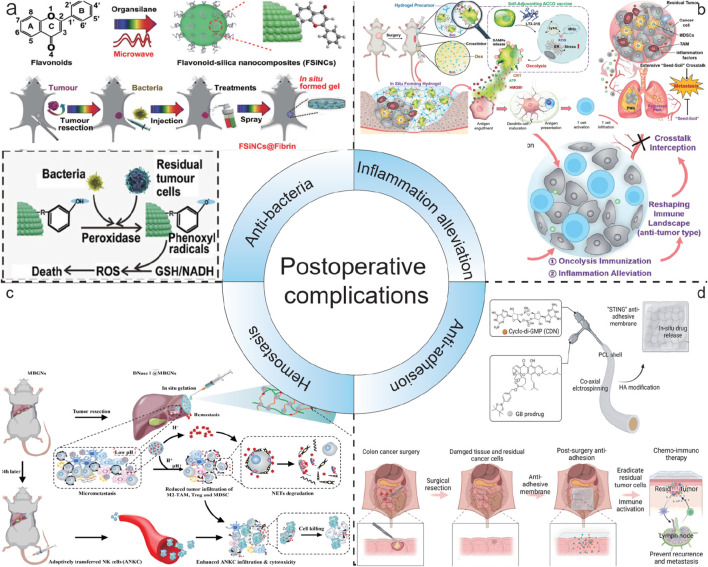
Schematic illustration of LDDS in postoperative complications. **(a)** Schematic illustration of FSiNCs-based nanohydrogel LDDS for anti-bacteria. Reprinted with permission from Ref (Chu et al., 2021). Copyright 2022 WILEY. **(b)** Schematic illustration of a trauma-inflammationresponsive and alleviating hydrogel scaffold LDDS for inflammation alleviation. Reprinted with permission from Ref (Li et al., 2022). Copyright 2022 American Chemical Society. **(c)** Schematic illustration of GODM-gel LDDS for swift hemostasis. Reprinted with permission from Ref (Cheng et al., 2022). Copyright 2022 Elsevier. **(d)** Schematic illustration of the fabrication process and the mechanism of action of the dual-drug loaded electrospun membrane LDDS for anti-adhesion. Reprinted with permission from Ref (Wang R. et al., 2024). Copyright 2024 WILEY.

Postoperative infection is the most prevalent complication following tumor surgery, and it is attributed to multiple factors (Sun and Kim, 2021). Initially, surgery can impair the skin and mucosal barriers of patients, offering an entry point for bacterial intrusion (Xia et al., 2022). Secondly, tumor patients typically have a lower immunity, making it challenging for them to effectively combat infections. Furthermore, the inappropriate use of antibiotics before and after surgery may result in the emergence of bacterial resistance, consequently augmenting the risk of infection. Thus, Hsiu-Mei Li’s group developed a novel multifunctional mesoporous bioactive glass (Fe-MBG–SS–CPT-FA@TC) that integrates chemotherapeutic, magnetothermal, chemodynamic, and antibacterial properties for targeted tumor therapy and postoperative infection prevention (Wei et al., 2024). Tetracycline (TC) was employed as the antibacterial agent, which was loaded into the pores of the multifunctional mesoporous bioactive glass (Fe-MBG). This allows for a sustained release of the antibiotic in the affected area following surgery to prevent bacterial infections. The study indicates that the sustained release of tetracycline from Fe-MBG–SS–CPT-FA@TC generate an antibacterial environment within the postoperative bone cavity. This can decrease the risk of infection. Additionally, the release of tetracycline can be controlled through the porous structure of Fe-MBG to meet various therapeutic needs. These characteristics indicate that Fe-MBG–SS–CPT-FA@TC is not only an effective material for tumor treatment but also possesses excellent antibacterial properties, which contribute to enhance surgical success rates and better quality of postoperative recovery for patients. Meanwhile, by introducing thrombin and fibrinogen, Yao He’s group resulted that FSiNCs@Fibrin gel was formed *in situ* at the tumor surgical bed to prevent postoperative tumor recurrence (Figure 13a). Compared with free counterparts, the locally released FSiNCs had an 18-fold enhancement in antibacterial effect and a 12-fold increase in antitumor effect *in vivo*. In addition, the previously mentioned LDDS combined with PTT can also inhibit the growth of bacteria and achieve the effect of preventing postoperative infection.

Postoperative inflammatory response is a common and crucial complication that cannot be ignored after tumor surgery (McSorley et al., 2016). During the surgical procedure for tumors, the body undergoes trauma, and the damaged tissues will initiate a series of immune reactions, consequently resulting in inflammation (Barhoum et al., 2023). This inflammatory response is typically a natural reaction of the body to the surgical wound. However, if it is not adequately controlled, it may adversely affect the patient’s recovery. Surgical trauma can aggravate the colonization of residual tumor cell “seeds” in the pre-metastatic niches (PMNs) “soil” at distant sites, thereby promoting postoperative metastasis. The inflammatory response plays a dual role after surgery by reshaping the local immune environment and resuscitating autologous cancer cells succumbing to oncolysis (ACCO) (Li et al., 2022). This helps to eradicate the residual tumor “seeds” and simultaneously intercept the “seed-soil” crosstalk, normalizing the distant lung and leading to the regression of the pre-existing PMNs “soil”. Lian Li designed an injection hydrogel *in situ* that can respond to the enriched ROS at the trauma site, enabling the local delivery and on-demand release of ACCO and anti-inflammatory agents (Figure 13b) (Li et al., 2022). They presented an innovative approach that effectively suppresses postoperative tumor recurrence and metastasis by combining oncolysis immunization and inflammation alleviation. Through the use of a trauma-responsive hydrogel scaffold, it achieves effective regulation of the inflammatory microenvironment, offering new perspectives for postoperative tumor treatment.

Postoperative bleeding is a common complication following tumor surgery, and it can happen at various stages after the operation, bringing potential risks to the patient’s recovery (Ohta et al., 2019). During the surgical procedure, although doctors will make every effort to take all kinds of measures to control the bleeding, it is sometimes hard to entirely prevent the occurrence of postoperative bleeding due to factors such as the location and size of the tumor, as well as the complexity of the surgery. Wenjie Chen reported a novel, dual pH-responsive adhesive hemostatic hydrogel (GODM-gel) (Figure 13c) (Cheng et al., 2022). GODM-gel offered rapid adhesion upon injection at the surgical margin, forming a stable gel that achieves swift hemostasis. Both *in vitro* and *in vivo* experiments demonstrated that the GODM-gel possessed superior adhesive strength and burst pressure compared to conventional hemostatic agents, such as fibrin glue, indicating its exceptional hemostatic capabilities in surgical procedures.

Tissue adhesion following tumor surgery is one of the common postoperative complications (Wang J. et al., 2024). After a tumor operation, the normal separating structure between the body’s tissues and organs is disrupted. In the healing process, tissues that should have been independent are joined by fibrous tissue, resulting in tissue adhesion. This connection can lead to various problems and requires careful management. Post-surgery tissue adhesion also limits the possibility of reoperation, affecting long-term survival of cancer patients (Wang J. et al., 2024). To address CRC recurrence and post-surgery tissue adhesion, Qingsong Yu’s group developed a novel stimulator of interferon genes (STING) membrane using coaxial electrospinning technology and hyaluronic acid modification (Figure 13d) (Wang R. et al., 2024). The membrane co-loads a ROS responsive prodrug of gambogic acid (GB) and a potent STING agonist (CDN), enabling sustained and sequential drug release. Localized delivery of GB and CDN can selectively induce efficient immunogenic cell death of cancer cells and activate the systemic anticancer immunity by stimulating the cGAMP synthase/STING pathway. The “STING” membrane not only prevents tumor recurrence through synergistic chemoimmunotherapy but also avoids post-surgery tissue adhesion, facilitating clinical intervention for CRC.

## 6 Conclusion and outlook

This review comprehensively summarizes the significant potential demonstrated by the current nanotechnology-based LDDS. It not only has a positive impact on the future development of tumor surgery, providing more effective adjuvant treatment modalities, but also significantly reduces the recurrence and metastasis of postoperative tumors, lowering the risk of tumor recurrence. Furthermore, LDDS shows outstanding capabilities in the treatment of various complications after surgery. It effectively alleviates the suffering of patients and improves their quality of life. Simultaneously, LDDS enhances the therapeutic effect. It contributes to prolonging the survival period of patients, providing them with more hope for survival.

However, in this field, there are still several issues. These issues require careful consideration and resolution:

1. Stability and Long-Term Storage: The LDDS need be stable and capable of long-term storage under various environmental conditions without losing its activity. This involves resistance to temperature, humidity, and light. Moreover, it requires maintaining the completeness of the system’s structure and function during storage.
2. Biocompatibility: The LDDS requires excellent biocompatibility. This is to minimize toxicity and immune reactions towards normal tissues.
3. Biosafety: The LDDS must demonstrate good biosafety. In other words, it should not lead to unexpected side effects or long-term adverse consequences within the body.
4. Drug Release Kinetics: The LDDS is required to have the ability to control the release rate of the drug. It also needs to control the release duration of the drug to ensure optimal therapeutic efficacy within the treatment window.
5. Clinical Translation: Translating laboratory research into clinical applications is a complex process. It requires overcoming multiple obstacles, such as obtaining regulatory approval, conducting cost - effectiveness analysis, and designing clinical trials.

In summary, LDDS are playing an increasingly crucial role in the treatment of postoperative complications of tumors. This system has the ability to release drugs directly at the surgical site after tumor resection, which effectively reduces the risk of tumor recurrence. Simultaneously, it exhibits remarkable efficacy in controlling postoperative complications, such as infection, inflammation, and adhesion. It not only improves the therapeutic outcome but also enhances the patients’ quality of life and decreases the incidence of postoperative complications. With the continuous progress of materials science and biomedical engineering, the future design of LDDS will be more intelligent and individualized to meet the diverse needs of clinical treatment.
